# Analysis of outcomes and reasons for refusing organs offered by the National Transplant Center

**DOI:** 10.31744/einstein_journal/2025AO1598

**Published:** 2025-07-30

**Authors:** Patricia Freire, Carmelia Matos Santiago Reis, Maria Rita Carvalho Garbi Novaes

**Affiliations:** 1 Fundação de Ensino e Pesquisa em Ciências da Saúde Brasília DF Brazil Fundação de Ensino e Pesquisa em Ciências da Saúde, Brasília, DF, Brazil.; 2 Fundação de Ensino e Pesquisa em Ciências da Saúde Escola Superior de Ciências da Saúde Brasília DF Brazil Escola Superior de Ciências da Saúde, Fundação de Ensino e Pesquisa em Ciências da Saúde, Brasília, DF, Brazil.

**Keywords:** Transplantation, Organ preservation, Tissue and organ procurement, Organ transplantation, Waiting lists, Refusal to participate

## Abstract

**Objective:**

To investigate the outcomes and reasons for the refusal of organs offered on a single national list managed by the National Transplant Center.

**Methods:**

This retrospective cohort study was conducted with a quantitative approach, using data from offers of organs not used in the states and the Federal District, but offered to the National Transplant Center for national distribution.

**Results:**

Of the 22,824 offers of unused organs in the states to the National Transplant Center, 8,483 (37%) were accepted and 14,341 (63%) refused. Of the accepted organs, 6,433 (76%) were implanted and 2,050 (24%) were not used.

**Conclusion:**

The use of organs at the interstate (national) level could be improved, and ascertaining the reasons for refusal could contribute to intervention strategies.

## In Brief

This study analyzed 22,824 organ offers made to Brazil's National Transplant Center. Of these, 37% were accepted and 63% were refused. Among the accepted organs, 76% were transplanted. Understanding reasons for refusal may help improve national organ use and guide the developmentof effective strategies.

## Highlights

■ Logistical factors were not responsible for most organ rejections in the single national waiting list.
■ Refusal information was operator dependent, as the reason for refusal was generic in most cases.
■ It is possible to increase the use of organs offered to the National Transplant Center by converting more offers into transplants performed through a more detailed study of the reasons for refusal.

## Graphical abstract

**Figure:**
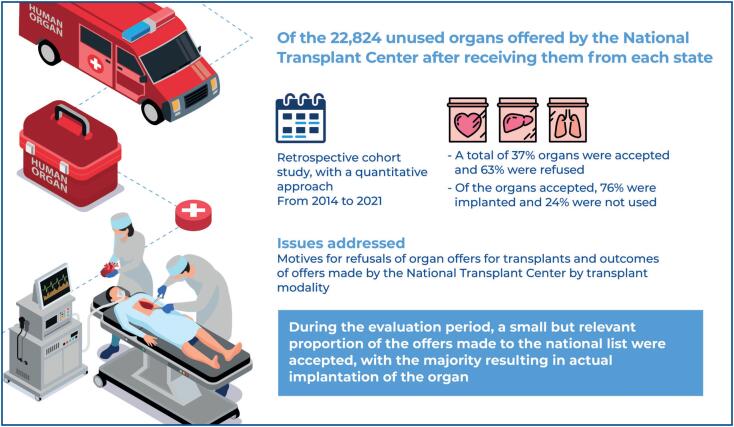

## INTRODUCTION

In Brazil, some organs are not used by states that do not perform a certain type of transplant or do not have suitable recipients to receive grafts and are refused after being offered by the National Transplant Center (CNT - *Centro Nacional de Transplantes*) of the Ministry of Health (MS - *Ministério da Saúde*). Understanding the reasons for refusal may decisively change the scarcity scenario and increase the availability of organs, especially considering the growing list of transplant candidates.^(1)^ Analyzing these reasons can reveal opportunities to optimize allocation to the national list and reduce the nonuse of viable organs for transplants.

The first human organ transplant dates back to the 1930s, when a Soviet surgeon grafted a kidney into a recipient with an incompatible ABO blood group.^(2)^ Improved knowledge of graft tissue rejection and immunological tolerance is essential to establish human organ transplantation as an effective therapeutic method for treating several terminal diseases.^(3)^

Access to organ transplants in Brazil is granted through inclusion in a single waiting list established under Law No. 9,434/1997.^(4)^ The criteria for allocating organs for transplantation in Brazil are defined in the MS regulations.^(5)^ The functions of the National Transplant System (SNT - *Sistema Nacional de Transplantes*) are exercised by MS through the General Coordination of the National Transplant System (CGSNT - *Coordenação Geral do Sistema Nacional de Transplantes*).^(6)^

In this context, it is important to understand how these organs are distributed to recipients. As defined in the presidential decree,^(6)^ a single transplant waiting list is organized based on regional, state, macroregional, and national lists. Thus, when an organ is offered for transplantation, a compatible recipient is first sought within the regional list organized in Federative Units. If no compatible recipient is identified, the organ is offered to the Federative Units list, which aggregates all potential recipients in the state (or Federal District). If the type of donor organ transplantation is not performed in the Federative Unit, or if no compatible recipient is found at the state level, the organ is offered to the national list, managed by the CNT.

**Figure 1:**
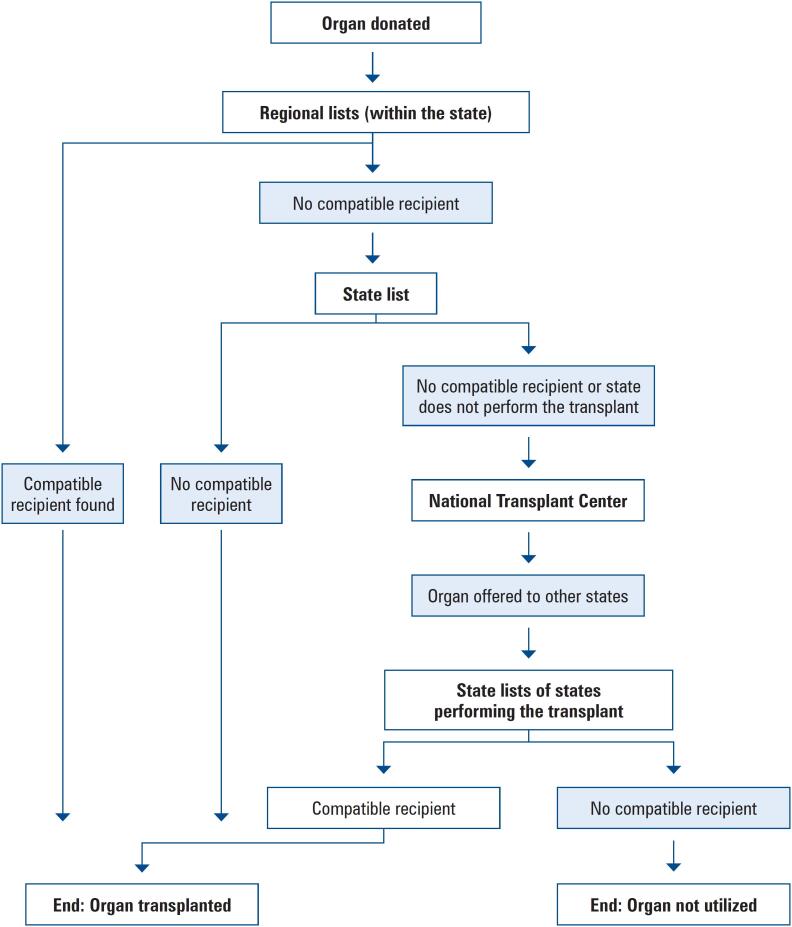
Flowchart depicting the distribution of organs in the states with the aid the national list of CNT/MS

The dynamics of the distribution of donated organs is represented by figure 1.

When organizing the list, the organ is also offered based on blood compatibility, severity, anthropometric correlation, waiting time on the list, and immunogenetic compatibility, in the case of the kidney.

The acceptance or refusal of organs is the responsibility of the transplant teams^(4)^ who assess technical conditions (gravity, cold ischemia time, age, etc.), structural conditions (availability of intensive care beds, existence of preservation fluid, etc.), and logistical conditions (existence of airport or landing strip, availability of flight compatible with cold ischemia time, etc.).

Organ refusal may be linked to several factors, including those related to the donors (infection, positive serology for communicable diseases, etc.), transport logistics (lack of flights, unfavorable weather conditions, etc.), conditions of the recipients (incomplete pretransplant exams, infection, etc.), conditions of the organ (poor perfusion, poor packaging, etc.), and other factors (cardiorespiratory arrest of the donor, family withdrawal, etc.).^(7)^

This paper describes the outcomes and reasons for the refusal of organs that were not used in the states owing to a lack of compatible recipients or because the transplant modality was not performed, and which are offered for inclusion in the single national list. The outcomes of the offers by organ type were also analyzed.

## OBJECTIVE

To analysis the outcomes and reasons for the refusal of organs offered within the scope of national allocation.

## METHODS

### Study design

This is a retrospective cohort study, with a descriptive nature and quantitative approach from 2014 to 2021.

### Location and period of research

The research was carried out in Brasília/DF using a dataset from the CNT of MS, which contained data on the outcomes and reasons for refusal of organs of agencies not used in the states but offered to the CNT. The data were made available in an Excel spreadsheet after the Integrated Ombudsman and Access to Information Platform (Fala. BR system) requested access based on the Access to Information Law (LAI) and were processed using the same application.

### Research stages

#### Inclusion criteria

The outcomes and reasons for refusal of offers of isolated hearts, lungs, livers, kidneys, and pancreas were analyzed.

#### Exclusion criteria

Offers of simultaneous kidney/pancreas, isolated intestine, and multivisceral organs in the block were excluded, because the method of recording these offers was not standardized and, in the case of multivisceral organs, because they were only recorded from 2019 onwards. Data from the years prior to 2014 were not computed as they were not systematized in the MS database and remained only in physical files that were not made available.

### Data collection and analysis

Based on the data collected using the CNT organ distribution registration instrument (in an Excel spreadsheet), the outcomes were divided into four general event groups (GEg): GEg1 – accepted organs, GEg2 – refused organs, GEg3 – implanted organs and GEg4 – unused organs.

General outcomes were also analyzed by organ offered, generating five event groups by organ type (GEor): GEor1 – type of organs accepted, GEor2 – type of organs refused, GEor3 – type of implanted organs, and GEor4 – type of unused organs.

The reasons for refusal were grouped into five event groups related to the following reasons for refusal (GEr): GEr1 – refusals due to logistics, GEr2 – refusals due to donor conditions, GEr3 – refusals due to recipient conditions, GEr4 – refusals due to agency conditions, and GEr5 – refusals for other reasons.

## RESULTS

The number of general outcomes GEg of offers made by the CNT totaled 22,824 organ offers from 2014 to 2021. Of these, 8,483 (37%) were accepted and 14,341 (63%) were refused. Of the total organs accepted, 6,433 (76%) were implanted and 2,050 (24%) were not used despite initial acceptance (Table 1).

**Table 1:** Total number and percentage of outcomes from 2014 to 2021 according to the Ministry of Health, Brasília, DF - Brazil

Variable	Total	GEg outcome groups			
		Accepted organs n (%)	Refused organs n (%)	Implanted organs n (%)	Unused organs n (%)
Year/total	22,824	8,483 (37)	14,341 (63)	6,433 (76)	2,050 (24)
2014	2,161	929 (43)	1,232 (57)	730 (79)	199 (21)
2015	2,165	808 (37)	1,357 (63)	628 (78)	180 (22)
2016	2,358	825 (35)	1,533 (65)	646 (78)	179 (22)
2017	2,929	1,090 (37)	1,839 (63)	824 (76)	266 (24)
2018	3,074	1,193 (39)	1,881 (61)	948 (79)	245 (21)
2019	3,442	1,272 (37)	2,170 (63)	860 (68)	412 (32)
2020	3,320	1,165 (35)	2,155 (65)	866 (74)	299 (26)
2021	3,375	1,201 (36)	2,174 (64)	931 (78)	270 (22)

Source: Brasil. Ministério da Saúde. Dados disponibilizados em planilha Excel, mediante solicitação ao sistema Fala. BR. [citado 2025 Jan 8]. Disponível em: https://sei.saude.gov.br/sip/login.php?sigla_orgao_sistema=MS&sigla_sistema=SEI^(7)^ GEr: general event groups.

Regarding the outcome by type of organ offered (GEor), 511 (16%) hearts, 212 (12%) lungs, 2,149 (37%) livers, 5,505 (54%) kidneys, and 106 (5%) pancreas were accepted. The refusals corresponded to 2,631 (84%) hearts, 1,559 (88%) lungs, 3,617 (63%) livers, 4,677 (46%) kidneys, and 1,857 (95%) pancreas. Regarding the effective implantation of the accepted organs, 441 hearts (86%), 164 lungs (77%), 1,738 livers (81%), 4,014 kidneys (73%), and 76 pancreas (72%) were implanted. Despite initial acceptance, 2,050 organs were not used. Of the total number of unused organs, 70 corresponded to the heart (14%), 48 to the lungs (23%), 411 to the liver (19%), 1,491 to the kidneys (27%), and 30 (28%) to the pancreas (Table 2).

**Table 2:** Type of organ and percentage of outcomes from 2014 to 2021 according to the Ministry of Health, Brasília, DF - Brazil

Variable	Total	GEor outcome group			
		Accepted organs n (%)	Refused organs n (%)	Implanted organs n (%)	Unused organs n (%)
Organ	22,824	8,483 (37)	14,341 (63)	6,433 (76)	2,050 (24)
Heart	3,142	511 (16)	2,631 (84)	441 (86)	70 (14)
Lung	1,771	212 (12)	1,559 (88)	164 (77)	48 (23)
Liver	5,766	2,149 (37)	3,617 (63)	1,738 (81)	411 (19)
Kidney	10,182	5,505 (54)	4,677 (46)	4,014 (73)	1,491 (27)
Pancreas	1,963	106 (5)	1,857 (95)	76 (72)	30 (28)

Source: Brasil. Ministério da Saúde. Dados disponibilizados em planilha Excel, mediante solicitação ao sistema Fala. BR. [citado 2025 Jan 8]. Disponível em: https://sei.saude.gov.br/sip/login.php?sigla_orgao_sistema=MS&sigla_sistema=SEI^(7)^ GEor: general organ.

Among the 14,341 organs refused, 850 (6%) were refused for logistical reasons (GEr), 8,530 (59%) for donor conditions, 652 (5%) for recipient conditions, 1,264 (9%) for organ conditions, and 3,045 (21%) for other unspecified reasons (Table 3).

**Table 3:** Total number and percentage of reasons for refusals per year (2014 to 2021) according to the Ministry of Health, Brasília, DF – Brazil

Variable	Total	Group of reasons for refusals, Ger				
		Logistics n (%)	Donor conditions n (%)	Receiver conditions n (%)	Organ conditions n (%)	Others n (%)
Year/total	14,341	850 (6)	8,530 (59)	652 (5)	1,264 (9)	3,045 (21)
2014	1,232	183 (15)	485 (39)	27 (2)	153 (12)	384 (31)
2015	1,357	232 (17)	564 (42)	25 (2)	159 (12)	377 (28)
2016	1,533	126 (8)	764 (50)	28 (2)	203 (13)	412 (27)
2017	1,839	104 (6)	1,052 (57)	44 (2)	224 (12)	415 (23)
2018	1,881	88 (5)	1,256 (67)	38 (2)	168 (9)	331 (18)
2019	2,170	42 (2)	1,410 (64)	115 (5)	118 (5)	485 (22)
2020	2,155	29 (1)	1,468 (67)	88 (4)	105 (5)	465 (21)
2021	2,174	46 (2)	1,531 (69)	287 (13)	134 (6)	176 (8)

Source: Brasil. Ministério da Saúde. Dados disponibilizados em planilha Excel, mediante solicitação ao sistema Fala. BR. [citado 2025 Jan 8]. Disponível em: https://sei.saude.gov.br/sip/login.php?sigla_orgao_sistema=MS&sigla_sistema=SEI^(7)^ GEr: general event groups.

## DISCUSSION

In Brazil, the demand for solid organ transplants (heart, lungs, kidneys, liver, and pancreas) is greater than their availability. Data from the MS shows that the waiting list had 34,830 patients by the end of 2022.^(1)^ In the same year, 7,473 organ transplants were performed, highlighting the disproportion between demand and supply.

In a scenario of insufficient organs on the transplant waiting list, knowing the outcome of the offers made by the State Transplant Centers to the CNT and the reasons why the organs were disregarded are of great importance, as refusals corresponded to 63% of the offers made during this period.^(7)^ The responsibility of the management of the SNT and the transplant community to increase the use of available organs from deceased donors increases the importance of investigating the outcomes of the offers to determine the causes of the large number of refusals and possibilities for intervention in the scenario.

Reasons for refusal may be related to the donor's clinical conditions (such as hemodynamic instability or severe infection) or even the unavailability of transplant teams on weekends. In this study, 59% of the organs offered were refused because of "donor conditions," and it was not possible to establish the specific reason for refusal. In contrast, a 2018 study showed that 36,700 (17%) of 212,305 kidneys from deceased donors made available for transplantation were refused due to anatomopathological findings between 2000 and 2015 in the United States of America (USA).^(8,9)^

Another 2018 study analyzed data regarding 3,863 kidneys refused by an Organ Procurement Organization from 2001 to 2006 in Southern California/USA and identified other factors for refusals, such as anatomical abnormalities (4.2%) and age (3.5%).^(10)^ Consistent with a 2014 study^(11)^ that showed that half of the kidneys from donors with expanded criteria were refused in the USA, the data from this study demonstrated that 46% of the kidneys were refused in Brazil, although it was not possible to establish the reason for the refusal of these organs.

The refinement of the reasons that lead to organ refusal in Brazil has not yet been explored, and the lack of standardization of these reasons may contribute to false conclusions. According to a survey conducted in the Computerized Waiting List Management System (SIG/SNT - *Sistema de Informações Gerenciais/Sistema Nacional de Transplantes*) of the MS, several established reasons for refusal have been described in a menu made available by the system itself (Table 4).

**Table 4:** Menu of reasons for organ refusals published by the SIG/SNT

Reasons for refusal	
Morphological changes	Altered blood gas analysis
Morbid background	Other donor characteristics
Absence of donor examinations	Other administrative
Absence of serology for cytomegalovirus and toxoplasmosis	Other administrative/donor
Donor's clinical conditions	Other administrative/receiver
Donor conditions	Positive serology
Distance/transport team	Distant receiver
Altered cardiac enzymes	Receiver not located
Team unavailable	Recipient needs double transplant
Team unavailable/combined surgery	Recipient without clinical conditions
Team unavailable/congress	Hypersensitized receptor/panel
Staff unavailable/vacation	Receiver did not appear in a timely manner
Unavailable team not located	Recipient refusal
Team did not respond within 1 hour	Denial of SARS-CoV-2 pandemic
Team/establishment with expired authorization	No contact with CNCDO
Steatosis	No bed for transplants
Altered exams	No preservation medium/surgical material
Fever/evidence of infection	No operating room available
Altered liver function	Inadequate size/weight for the receiver
Recovered liver function	Distance/transportation team
High blood pressure	Prolonged ischemia time
Donor age	Transplant with another donor
Traumatic organ injury	Transplant recipient prioritized state
Vascular injury	Liver transplant
Clinical improvement of the recipient	Use of hepatotoxic drugs
Receiver improvement	Use of vasopressor drugs
Not offered/donor from another macroregion	Cross match not performed
Death of the recipient	Positive cross match
Altered catheterization	Absence of tacrolimus dosage
Catheterization not performed	High creatinine
Altered ECG	Nephrectomy/altered biopsy
Altered echocardiogram	Recipient refusal
Echocardiogram not performed	Denial of SARS-CoV-2 pandemic
Altered cardiac enzymes	SARS-CoV-2 positivity
Donor cardiac arrest	No contact with CNCDO
Altered chest x-ray	No bed for transplants

Source: Brasil. Ministério da Saúde. Dados disponibilizados em planilha Excel, mediante solicitação ao sistema Fala. BR. [citado 2025 Jan 8]. Disponível em: https://sei.saude.gov.br/sip/login.php?sigla_orgao_sistema=MS&sigla_sistema=SEI^(7)^ CNCDO: *Centrais de Notificação, Captação e Distribuição de órgãos*.

The diversity of reasons and the lack or existence of duplicate reasons in the SIG/SNT contribute to the collection of inconsistent data. Moreover, the possibility of personal interpretation of the reasons during selection may contribute to contradictions in the results. In addition, the state of São Paulo uses a system that is different from that used by the MS, with a different menu of reasons for refusal (Table 5).

**Table 5:** Menu of reasons for organ refusals published by the SIG/SP

Distant receiver	Drug user/tattoo
Recipient without clinical conditions	High blood pressure
Receiver not located	Prolonged hospital stay
Recipient refusal	Use of vasopressors
Dialysis team/center not found	Hemodynamic instability
No operating room available	Prolonged intubation time
No surgical material/blood products	Morbid antecedents
Lack of preservation liquid	Age
Recovered liver function	Inadequate size/weight for the receiver
Incomplete recipient examinations	ABO non-equality
Receiver improvement	Inadequate preservation liquid
Distance/team transportation	Low HLA compatibility (donor × recipient)
Needs multiple organ transplant	Use of hepatotoxic drugs
Transplanted with another donor	Fever/evidence of infection
Inactive receiver	Donor cardiac arrest
Split liver transplant	Expanded donor criteria
No bed for transplant	Other-donor characteristics
Team did not respond within 1 hour	Lack of preservation liquid
Death	Hospital infection
Team unavailable	Received transfusion
Staff unavailable/other procedure	Hypersensitivity
Team unavailable/congress	Cross match not performed
Staff unavailable/vacation	Receiver/team refusal - pandemic
Team unavailable/combined surgery	Other-administrative/receiving

Source: Brasil. Ministério da Saúde. Dados disponibilizados em planilha Excel, mediante solicitação ao sistema Fala. BR. [citado 2025 Jan 8]. Disponível em: https://sei.saude.gov.br/sip/login.php?sigla_orgao_sistema=MS&sigla_sistema=SEI^(7)^

The lack of details regarding the reasons for refusal recorded by the CNT may have obscured important information. Our study findings agree with those of a study conducted in 2000 in the USA^(12)^ showing that more refusals were related to the conditions of the donor (59%) than those of the recipient (5%).

Nevertheless, the grouping of outcomes and reasons for refusal by the CNT allowed for important findings. For example, contrary to expectations, refusals due to "logistics" corresponded to the second lowest percentage of refusals in an eight-year period (6%), losing only to the reasons in the group "recipient conditions" (5%) in the same period.

It should be noted that within the scope of the CNT, the term "organ disposal" has a different connotation from the term "organ refusal." The first is used when an organ, after having already been collected, is destined for a pathological anatomy service (disposal) for justified reasons. The second is used to designate the refusal of an offer, even if the organ has not been removed (*i.e*., it remains in the anatomical cavity of the donor).

This study had limitations regarding data analysis, as the record of reasons for refusal used only data related to the predominant reasons. This is because the same organ can be offered more than once to the same transplant center. This recording method can contribute to the loss of important information because it fails to capture possible organ refusals motivated, for example, by management problems and weekends.

The absolute number and percentage of offers, acceptances, and refusals from 2014 to 2021 indicates that 22,824 of the 141,575 organ offers for transplant lists were made to the CNT/MS, which means that 16% of the offers were not exhausted in the states and continued to be offered to the national list managed by the CNT/MS. Most of the organs were distributed within the states themselves, comprising 84% of the offers concluded within the states’ territory without being included in the national list (Table 6).

**Table 6:** Absolute number and percentage of offers, acceptances, and refusals by year (2014 to 2021) according to the Ministry of Health, Brasília, DF - Brazil

Total offers	Accepted		Refused		Total	
	(n)	%	(n)	%	(n)	%
State lists	47,567	85	71,184	83	118,751	84
National list	8,483	15	14,341	17	22,824	16
Total	56,050	100	85,525	100	141,575	100

Source: Brasil. Ministério da Saúde. Dados disponibilizados em planilha Excel, mediante solicitação ao sistema Fala. BR. 2022 [citado 2025 Jan 8]. Disponível em: https://sei.saude.gov.br/sip/login.php?sigla_orgao_sistema=MS&sigla_sistema=SEI^(7)^

Of the 22,824 offers on the national list, 63% were refused. Thus, although most organ acceptance occurs in the supplying states (84%), a significant portion is offered to the CNT/MS but not used for transplantation (63% refusal rate within the national list). Changing the scenario of refusal within the national list could contribute to reducing waiting lists in states.

## CONCLUSION

During the evaluation period, a small but relevant proportion of the offers made to the national list were accepted, with the majority resulting in actual implantation of the organ. Nonuse occurred in a proportion of the outcomes in the accepted organ group. Regarding organ type, the kidneys had the highest acceptance rate, followed by the liver. In contrast, the highest refusal rate was observed in the lungs, followed by the pancreas. The heart had the highest implantation rate, followed by the pancreas and kidneys. Surprisingly, a small proportion of refusals was attributed to logistics, while the majority were due to donor conditions, unspecified reasons, organ conditions, or recipient conditions.

Increasing the supply of organs and reducing the waiting time for transplantation requires a deeper understanding of the reasons for organ refusal in Brazil. The current system for recording refusals does not capture these reasons in detail, thus limiting our understanding of the reasons for refusals. Determining the causes of rejection can help in developing strategies that improve organ use. The clinical conditions of the donors were the main causes of rejection, highlighting the need for interventions that address these issues, from standardizing the reasons for rejection of professional training to reducing avoidable losses. In the future, a computerized system that manages waiting lists should have reliable reports that allow for better analysis of the data on the acceptance and rejection of organs offered for transplantation, not only for the academic community but also for managers and other members of the SNT.
